# Effect of the Membrane Composition of Giant Unilamellar Vesicles on Their Budding Probability: A Trade-Off between Elasticity and Preferred Area Difference

**DOI:** 10.3390/life11070634

**Published:** 2021-06-29

**Authors:** Ylenia Miele, Gábor Holló, István Lagzi, Federico Rossi

**Affiliations:** 1 Department of Chemistry and Biology “A. Zambelli”, University of Salerno, Via Giovanni Paolo II 132, 84084 Fisciano, SA, Italy; ymiele@unisa.it; 2 MTA-BME Condensed Matter Physics Research Group, Budapest University of Technology and Economics, Budafoki ut 8, 1111 Budapest, Hungary; hollo88@gmail.com; 3 Department of Physics, Budapest University of Technology and Economics, Budafoki ut 8, 1111 Budapest, Hungary; 4 Department of Earth, Environmental and Physical Sciences—DEEP Sciences, University of Siena, Pian dei Mantellini 44, 53100 Siena, Italy

**Keywords:** vesicles, division, urea–urease enzymatic reaction, bending modulus, budding, ADE theory

## Abstract

The budding and division of artificial cells engineered from vesicles and droplets have gained much attention in the past few decades due to an increased interest in designing stimuli-responsive synthetic systems. Proper control of the division process is one of the main challenges in the field of synthetic biology and, especially in the context of the origin of life studies, it would be helpful to look for the simplest chemical and physical processes likely at play in prebiotic conditions. Here we show that pH-sensitive giant unilamellar vesicles composed of mixed phospholipid/fatty acid membranes undergo a budding process, internally fuelled by the urea–urease enzymatic reaction, only for a given range of the membrane composition. A gentle interplay between the effects of the membrane composition on the elasticity and the preferred area difference of the bilayer is responsible for the existence of a narrow range of membrane composition yielding a high probability for budding of the vesicles.

## 1. Introduction

One of the most ambitious goals of the recently born discipline systems chemistry is the creation of synthetic life following a bottom-up approach [1,2,3,4]. Such research and investigations are important for the design of stimuli-responsive materials and their usage in several applications (e.g., targeted drug delivery) [5,6] and to understand fundamental open questions in the origin of life studies [2,7]. A general approach followed in this context is to recreate life-like functions within a minimal artificial cell-like container (water-in-oil droplets, emulsions, protocells, etc.) rather than replicate the complex biological environment typical of the living cells [8,9]. Several attempts have been made to imitate distinctive processes that characterize modern cells, such as intercellular communication [10,11,12,13,14], energy harvesting [15,16], metabolism [17,18] and self-replication or division [19,20,21,22,23,24,25,26,27,28,29].

The most common model for the investigation of the division and budding processes are giant unilamellar vesicles (GUVs) stabilized by an amphiphilic bilayer made of phospholipids and/or fatty acids. In general, division occurs when the volume of the vesicles decreases (mostly due to an osmotic shock) and concurrently the inner surface area of the inner bilayer leaflet decreases in respect to the outer one. Recently, we reported that a pH change occurring inside the lumen of mixed 1-palmitoyl-2-oleoyl-*sn*-glycero-3-phosphocholine (POPC)/oleic acid (HOA) GUVs can be utilized to drive and govern the division process [30,31]. In particular, the urea–urease enzymatic reaction [32,33,34] inside the lumen of the vesicles, coupled to the cross-membrane transport of urea, can trigger the shape deformation of the pH-sensitive mixed membrane by promoting the solubilization of oleate molecules in the inner water phase. In the framework of the Area Difference Elasticity (ADE) theory [23,35,36,37,38,39,40,41,42,43] we demonstrated that the shape deformation is driven by the synergic action of osmosis, acting on the volume of the vesicle, and pH change, the latter responsible for the increase of the preferred area difference of the bilayer leaflets. However, the ADE model predicts that the equilibrium shape of an elastic membrane undergoing a transformation, also depends on a third factor: the mechanical properties of the bilayer accounted by the ratio of the local (intrinsic fluidity of the membrane) and the non-local (tension generated by the different curvature of the two leaflets) bending moduli. In this study, we investigate the effect of the membrane composition on the division probability of the mixed GUVs. We present some experiments where we varied the total amphiphiles concentration (*s* = [POPC] + [HOA]) and the relative ratio between the two components (α = [HOA]/[POPC]) to show that α has an important role in determining the final shape of the vesicles. A general finding of this work is to assess the role of the mechanical properties of the membrane, such as the elasticity and the bending modulus, in driving the protocells division.

## 2. Materials and Methods

### 2.1. Experimental

For GUVs preparation and encapsulation of chemical species, the phase transfer method [33,44,45,46] was used, which allowed full encapsulation of chemical species. Briefly, the method consists in the preparation of two isotonic solutions: outer solution (O-solution) and inner solution (I-solution) separated by an interface made of amphiphilic molecules. The outer solution includes water and glucose (Sigma Aldrich, St. Louis, Missouri, USA), above this phase a mixture of 1-palmitoyl-2-oleoyl-*sn*-glycero-3-phosphocholine, POPC (Lipoid, Ludwigshafen, Germany) and oleic acid, HOA (Sigma Aldrich) in mineral oil (M5904 Sigma Aldrich) is allowed to incubate to make a monolayer. The inner solution is prepared pipetting by hand a water in oil microemulsion. The aqueous phase of the inner solution is composed of the enzyme urease (Sigma Aldrich, typical activity 34310 U/g), sucrose (Sigma Aldrich), the fluorescence probe pyranine (Sigma Aldrich) and water, while the apolar phase contains POPC and HOA in mineral oil (we used the same concentration of interface). After the period of incubation, the water in oil microemulsion is poured over the interface and centrifuged. The formation of the vesicles takes place at the interface of the two phases, where emulsion droplets sink by gravity in the outer phase and are coated by the amphiphilic molecules to form bilayers (sucrose and glucose are used to enhance the difference of density between the droplets and the outer solution). Details are reported in SI.

The lipid content in vesicles samples was determined by an HPLC apparatus (Agilent 1200 Infinity) by using a C18 column (Agilent Zorbax Eclipse Plus, 4.6×100 mm, 3.5 μm) and a mobile phase composed of acetonitrile, methanol and formic acid at 0.1% (all solvents from Sigma-Aldrich) in the proportions 50:45:5 (isocratic mode) [47]. HPLC experiments were done in three replicates by collecting together 4 pellets at a time. The yield was calculated as the average mass of POPC in a pellet over the total mass of POPC used in a typical preparation prior the vesicles formation.

### 2.2. Numerical Calculation

#### 2.2.1. Surface Evolver

In the area difference elasticity model [40,43] a vesicle is considered as an elastic membrane and its equilibrium shape can be found with the minimization of the Helfrich energy [48,49] (*W*). In this model not just the local principal curvatures ($C_{1}$ and $C_{2}$) can differ from their equilibrium value, i.e., from the local spontaneous curvature ($C_{0}$), but an elastic membrane is taken into account, so the area difference between the inner and outer leaflets (ΔA) can be different from its equilibrium value (from the preferred area difference $\Delta A_{0}$) as well. The relative significance of these terms can be fine tuned with the local and the non-local bending moduli ($\kappa_{c}$ and $\kappa_{r}$). The energy can be expressed as

(1) $$W=\frac{1}{2}\kappa_{c}\oint_{S}{\left(C_{1}+C_{2}-C_{0}\right)}^{2}dA+\frac{\kappa_{r}}{2A_{0}h^{2}}{\left(\Delta A-\Delta A_{0}\right)}^{2}$$

where $A_{0}$ is the area of the vesicle, *h* is the distance between the neutral surfaces of the leaflets and the integral is calculated on the surface of the vesicle (*S*) [35,36]. The area difference can be calculated from the integral of the mean curvatures

(2) $$\Delta A=h\oint_{S}^{}\left(C_{1}+C_{2}\right)dA$$

and ΔA values can be determined experimentally from images as well [30,31].

For simplicity, the energy of the vesicle is often expressed in a reduced form which can be observed after the division with the bending energy of the sphere ($8\pi \kappa_{c}$)

(3) $$w=\frac{1}{4}\oint_{S}{\left(c_{1}+c_{2}\right)}^{2}da+K{\left(\Delta a-\Delta a_{0}\right)}^{2}$$

where $\Delta a=\Delta A/8\pi hR_{s}$ is the reduced area difference, $\Delta a_{0}=\Delta A_{0}/8\pi hR_{s}$ and $R_{s}$ is the radius of the sphere. $K=\frac{\kappa_{r}}{\kappa_{c}}$ represents the relative weight of the energy terms and it represents an intrinsic property of the membrane, the other transformed variables are $da=dA/4\pi R_{s}$, $c_{1}=C_{1}R_{s}$, $c_{2}=C_{2}R_{s}$.

The energy minimization was performed with Surface Evolver (SE) 2.70 [50]. During the minimization the reduced volume

(4) $$\nu =\frac{V}{4/3\pi R^{3}}$$

was constrained where $R=\sqrt{A/4\pi }$ is defined as the radius of an equivalent sphere and *A* is the surface of the vesicle.

The shape of the vesicle is determined by three variables ($\Delta a_{0}$,ν,*K*) in the energy minimization. During the simulation—besides the shape of the vesicle—the value of the reduced area could be changed, since an elastic membrane is considered so it can be different from $\Delta a_{0}$. The value of the reduced volume (0.7) was determined experimentally and it was kept constant in every simulation and *K* was adjusted according to a given bilayer composition.

#### 2.2.2. ODE Model

To describe the pH change inside the GUVs and obtain $\Delta a_{0}$ in time induced by the urea–urease enzymatic reaction, we developed a seven chemical species kinetic model, which can be represented mathematically by a set of ordinary differential equations (ODEs)

(5) $$\frac{d[X]}{dt}=r(\left[X]\right)+k_{X}\left({\left[X\right]}_{o}-\left[X\right]\right)$$

where [X] denotes the concentration of the chemical species X, *r*([X]) represents the set of reaction rates involving X, and [X]${}_{o}$ is the concentration of the chemical species in the outer phase. The transfer rate $k_{X}$ (s${}^{-1}$) is proportional to the surface-to-volume ratio of the vesicle and to the specific membrane permeability of the species.

The change of the number of molecules of oleate in the inner and outer leaflets is linked to the pH dynamics described by (5) through the following differential equations

(6) $$\begin{array}{cc} \frac{dN_{\mathrm{outer}}}{dt} & =\frac{dN_{\mathrm{outerHOA}}}{dt}+\frac{dN_{{\mathrm{outerOA}}^{-}}}{dt} \end{array}$$

(7) $$\begin{array}{cc} \frac{dN_{\mathrm{outer}}}{dt} & =-k_{f}\left(N_{\mathrm{outer}}-N_{\mathrm{inner}}\frac{R_{s}^{2}}{{\left(R_{s}-h\right)}^{2}}\right) \end{array}$$

(8) $$\begin{array}{cc} \frac{dN_{\mathrm{inner}}}{dt} & =\frac{dN_{\mathrm{innerHOA}}}{dt}+\frac{dN_{{\mathrm{innerOA}}^{-}}}{dt}\\ \frac{dN_{\mathrm{inner}}}{dt} & =+k_{f}\left(N_{\mathrm{outer}}-N_{\mathrm{inner}}\frac{R_{s}^{2}}{{\left(R_{s}-h\right)}^{2}}\right) \end{array}$$

(9) $$\begin{array}{cc} \; & -N_{{\mathrm{innerOA}}^{-}}\;\frac{k_{\mathrm{off}}}{1+e^{\left(-k_{t}\;\left(\mathrm{pH}-{\mathrm{pH}}_{\mathrm{thres}}\right)\right)}} \end{array}$$

where $N_{\mathrm{inner}}$ and $N_{\mathrm{outer}}$ represent the total number of molecules (POPC + HOA + OA${}^{-}$) in the inner and outer leaflet, repsectively; $N_{\mathrm{innerHOA}}$ and $N_{{\mathrm{innerOA}}^{-}}$ indicate the number of oleic acid molecules (unionized and deionized form) present in the inner leaflet, $N_{\mathrm{outerHOA}}$ and $N_{{\mathrm{outerOA}}^{-}}$ are the oleic acid molecules in the outer leaflet; $k_{f}$ is the flip-flop constant for HOA and $k_{\mathrm{off}}$ is the kinetic constant for the solubilization of OA${}^{-}$; $R_{s}$ is the radius of the initial spherical vesicle, *h* is the distance between the two neutral surfaces. The number of molecules of POPC is considered constant. The logistic function in the last term of Equation (9) accounts for the pH-dependent formation of oleate aggregates in the vesicles lumen when a critical pH is reached (pH${}_{\mathrm{thres}}$).

The preferred area difference ($\Delta A_{0}$) and the normalized preferred area difference ($\Delta a_{0}$) were finally calculated in time as

(10) $$\Delta A_{0}\left(t\right)=N_{\mathrm{outer}}\left(t\right)<\tilde{a}>-N_{\mathrm{inner}}\left(t\right)<\tilde{a}>$$

(11) $$\Delta a_{0}\left(t\right)=\frac{\Delta A_{0}\left(t\right)}{8\pi R_{0}\left(t\right)h}$$

where $<\tilde{a}>$ is the average cross section of the amphiphiles and $R_{0(t)}=\sqrt{A_{0(t)}/\left(4\pi \right)}$ [23].

The model was described in details in ref. [31] and a short description can be found in the Supplementary Information (SI).

## 3. Results and Discussion

In a typical experiment, a fast enzymatic reaction inducing a pH change inside the GUVs with the consequent division of the vesicles, was obtained in the following conditions: urease (0.5 U/mL), pyranine (5.0 $\times \;10^{-5}$ M) and acetic acid (1 $\times \;10^{-6}$ M) were encapsulated in hybrid giant POPC/HOA vesicles with various amphiphiles total concentration [POPC]_0_ + [HOA]_0_ (*s*) and proportions [HOA]_0_/[POPC]_0_ (α). A urea solution ([CO(NH_2_)_2_]_0_ = 6.0 $\times \;10^{-2}$ M) was added to the vesicles suspension to initiate the reaction. Urea penetrated through the membrane of GUVs and initiated the urea–urease enzymatic reaction, which caused a pH increase (from ∼6 to ∼6.5, measured through the fluorescence of the pH-sensitive probe pyranine encapsulated in the vesicles lumen [30]) due to the produced ammonia. A shift towards basic conditions inside the vesicles lumen causes the deprotonation of HOA to form oleate molecules readily dissolved in the aqueous medium. In a previous work [31], we demonstrated that the addition of urea in the outer solution generates an hypertonic condition, which, in turn, drives a deflation of the vesicles due to the osmotic shock. Thus, the addition of urea triggers a synergistic dynamics between the reduction of the vesicles volume (ν changes from 1 to ∼0.7 at s=5 mM and α∼1) and the increase of the area difference between the lipid leaflets in the bilayer, caused by the dissolution of oleate molecules in the aqueous lumen ($\Delta a_{0}$ changes from 1 to ∼1.7 at s=5 mM and α∼1). According to the ADE theory the final values of ν and $\Delta a_{0}$ correspond to a budded shape of the vesicles in our experimental conditions.

After spanning different compositions of the membranes, we identified s=5 mM and α=0.92 ([HOA] = 2.4 mM, [POPC] = 2.6 mM) as the optimal condition to obtain the highest probability of successful budding. Actually, the budding rate was found substantially independent from the parameter *s* (∼16% in the interval 0.3 mM < *s* < 10 mM, (see Figure S1a in SI), while α was found to be determinant. This behaviour can be expected taking into account that the yield of the droplet transfer method, i.e., the mass of amphiphiles in a vesicles pellet over the total mass of amphiphiles initially present in the Eppendorf tube (see SI for preparation details), is substantially independent from *s* as revealed by HPLC analysis (see Figure S1b in SI); as previously reported [51], the total quantity of amphiphiles has a major impact on the number of generated vesicles rather then on their properties (size, relative composition, etc.).

**Figure 1:**
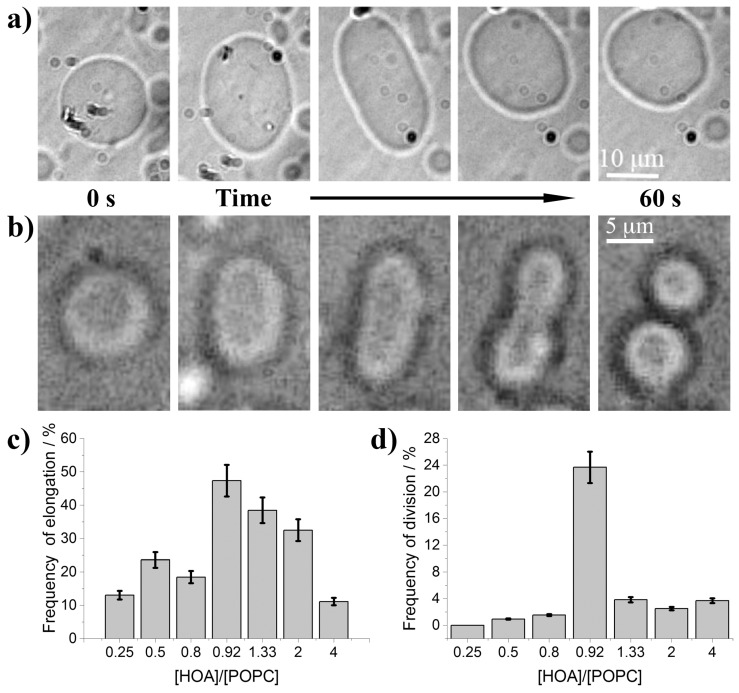
(**a**) Reversible shape transformation of a GUV from sphere to prolate and back to spherical shape triggered by the urea–urease enzymatic reaction, *s* = 5 mM, α = 0.5; (**b**) shape transformation of GUV leading to a final budded conformation, *s* = 5 mM, α = 0.92; (**c**) frequency of elongation as a function of the ratio [HOA]${}_{0}$/[POPC]${}_{0}$, *s* = 5 mM; (**d**) frequency of budding as a function of the ratio [HOA]${}_{0}$/[POPC]${}_{0}$, *s* = 5 mM.

In contrast, the relative composition of the membrane, α, impacted both the frequency of simple elongations (vesicles that evolved in a prolate or oblate shape but did not bud at the end of the process, Figure 1a,c) and the frequency of successful budding (Figure 1b,d). α was varied between 0.25 and 4 for a total amount of amphiphiles at fixed *s* = 5 mM and the ratio α = 2.4 mM/2.6 mM = 0.92 was found to correspond to the best budding rate, lower ratios or higher ratios are less effective. At a first glance, the optimal composition α = 0.92 might be considered a good compromise between the pH sensitivity given by the oleic acid and the stability guaranteed by POPC. In fact, high content of POPC made the vesicles less sensitive to pH changes and less prone to bud, in contrast high amounts of oleic acid might act as a buffer and interfere with the enzymatic reaction, though the reduced preferred area difference must in principle increase being more deprotonable molecules in the inner leaflet.

Therefore, to fully understand the effect of α, we should also consider as important parameter the elasticity of the membrane, expressed by the ratio of the local and non-local bending moduli *K* and determined by the composition of the membrane. In general, in mixed HOA/phospholipids membrane, the local bending rigidity ($\kappa_{c}$) increases whilst the non-local elastic modulus ($\kappa_{r}$) decreases with the oleic acid content, as found for 1,2-dioleoyl-*sn*-glycero-3-phosphocholine (DOPC)/HOA [52] and 1,2-dipalmitoyl-*sn*-glycero-3-phosphocholine (DPPC)/HOA [53] mixtures.

**Figure 2:**
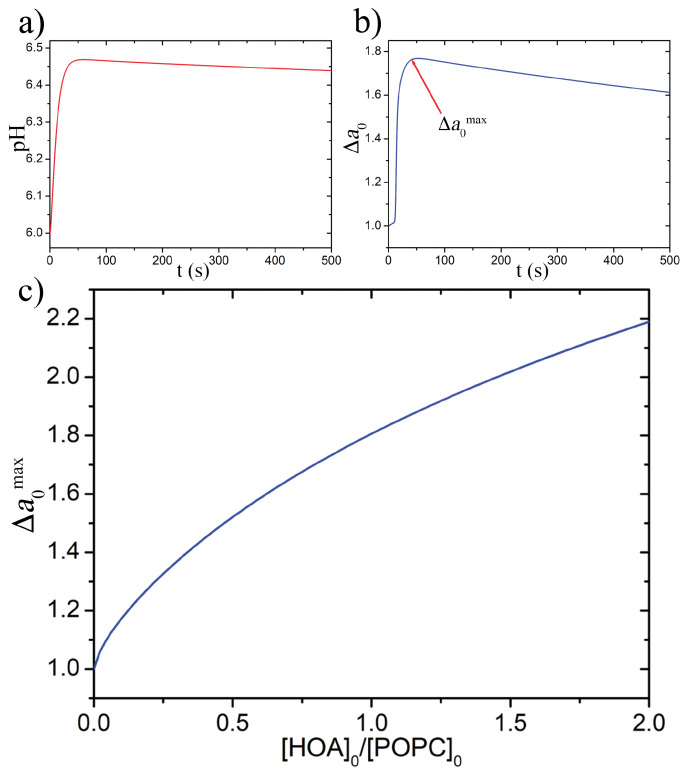
Examples of time course simulations of (**a**) pH and (**b**) $\Delta a_{0}$ inside a vesicle with [urease]${}_{0}$ = 1.1 U/mL, [CH_3_COOH]_0_ = $1\times 10^{-6}$ mol/L, [urea]${}_{0}$ = 60 mmol/L, *s* = 5 mmol/L and α = 1; (**c**) dependence of the maximum values of $\Delta a_{0}$ on the membrane composition, for each simulation at different α initial reactants concentration and *s* were kept constant.

To gain more insight into the dependence of the equilibrium limiting shapes of a vesicle, and hence the budding probability, on the membrane composition, we studied the influence of α on two parameters that drive the minimization of the energy functional *W*: $\Delta a_{0}$, which depends on the pH and *K*, which accounts for the elasticity of the membrane. The reduced volume ν was fixed to 0.7 since it was not influenced by α but only by the osmolarity difference between the lumen of a vesicle and the outer solution. The pH course inside a GUV and the variation of $\Delta a_{0}$ were calculated using the ODE model reported in the SI, running a time-course simulation (Figure 2a,b) for each α value in the interval 0.3–1.8, i.e., the one comprising the highest budding probability. From each simulation was then extracted the maximum value reached by $\Delta a_{0}$ ($\Delta a_{0}^{\max}$) which was finally plotted as a function of α (Figure 2c). As expected, despite a buffering effect of HOA, the net result of adding deprotonable molecules in the membrane is to increase $\Delta a_{0}^{\max}$.

To account for the dependence of *K* on the membrane composition we defined an arbitrary linear function, K=5−2.5α, which reflects the fact that by increasing the molecules of oleic acid the combined effect on the bending moduli causes *K* to decrease. Thus, *K* varies in the interval 4.25–0.85 that represents a reliable range for phospholipids-based bilayers [23,40,54].

**Figure 3:**
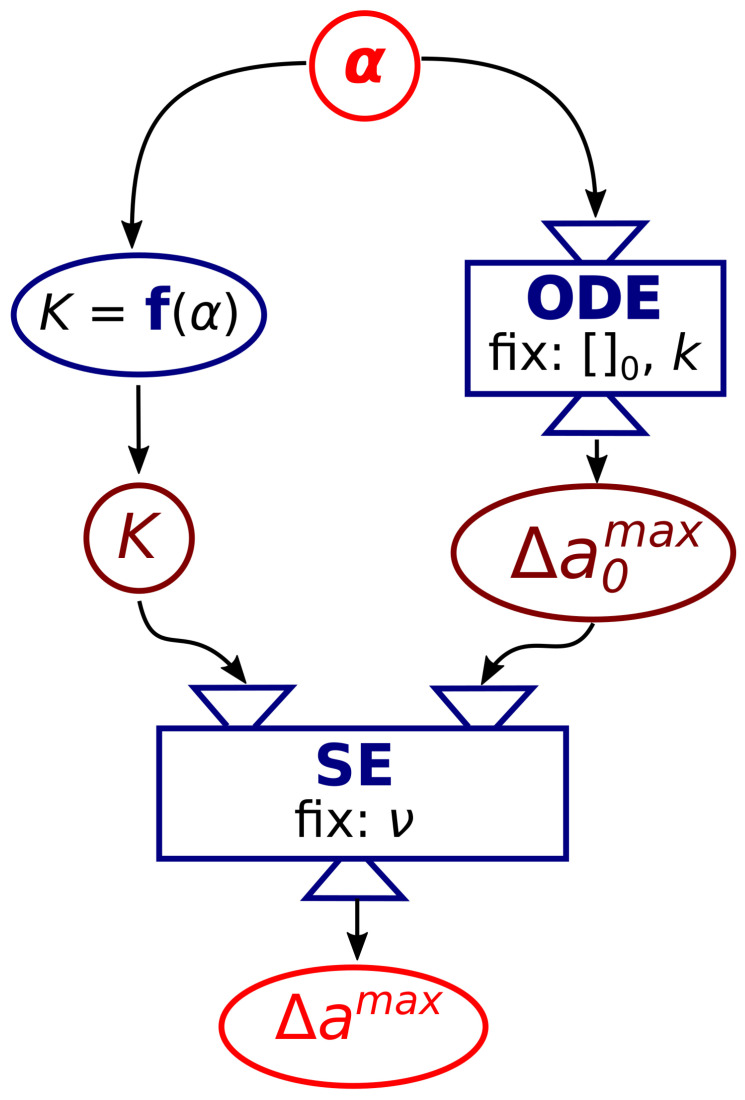
Sketch of the numerical simulations strategy. The membrane composition parameter, α, is used to calculate the corresponding elasticity ratio *K* through the linear empiric function K=f(α) and the value of $\Delta a_{0}^{\max}$ through the ODE model with fixed initial concentrations of reactants ([]${}_{0}$) and kinetic constants (*k*) for each run; *K* and $\Delta a_{0}^{\max}$ are then used in SE with fixed ν to yield the maximum reduced area difference $\Delta a^{\max}$.

According to the experimental observations, the budding limiting shape is a compromise between the increase of the reduced preferred area difference caused by the pH increase, with the consequent deprotonation of the inner leaflet, and an optimal elasticity of the membrane. We then used the values of *K* and $\Delta a_{0}$ as parameters in SE software to simulate the equilibrium limiting shape of a vesicle according to the methodology sketched in Figure 3. At a given α, the corresponding $\Delta a_{0}^{\max}$ and *K*, were calculated and used as input in SE together with the fixed ν.

The minimization of the energy functional *W* yields the reduced area difference $\Delta a^{\max}$ as output parameter, which, together with the fixed ν, determines the shape of the vesicle. According to the ADE theory, for ν=0.7 the corresponding value of Δa for the equilibrium budded shape has to be ∼1.4 [36,38,40,42].

**Figure 4:**
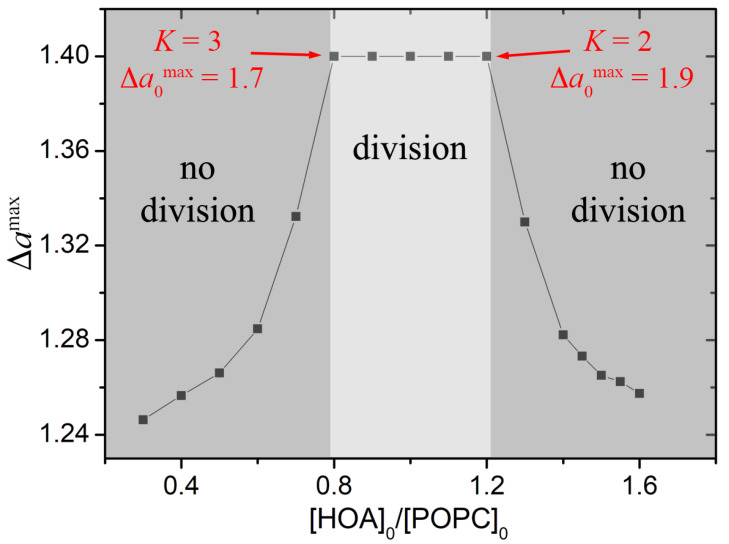
Phase diagram $\Delta a^{\max}$ – α showing the effect of the membrane composition on the successful budding for a vesicle, calculated through Surface Evolver software.

Figure 4 reports the phase diagram $\Delta a^{\max}$ – α where the output values of the energy minimization procedure are reported for each membrane composition. Simulations confirmed that a successful budding takes place when the membrane composition lies in a small interval around α=1 (HOA:POPC = 1:1); this represents the best trade off between the mechanical properties of the membrane and the capability of the enzymatic reaction to increase the preferred area difference. When α < 0.8 the deprotonated molecules in the inner leaflet are not enough to bring the vesicle to a budded shape. In contrast, when α > 1.2, the enzymatic reaction causes the dissolution of a high amount of oleate but the membrane oppose more resistence to be effectively deformed.

## 4. Conclusions

The division process is one of the fundamental function necessary for the self-replication and self-reproduction of biological entities. In modern cells replication is driven by a complex mechanism of growth and division, regulated by proteins and involving several chemico-physical dynamics (mass transfer, phospholipids synthesis, enzymatic reactions, etc.) [55,56]. However, in the context of abiogenesis, to understand the basic mechanisms at the basis of protocells division can be helpful to look for the simplest chemical and physical process likely at play in prebiotic conditions. For a few years our group has been studying a simple cell model having a diameter larger than 5 μm (GUV) and based on a mixed phospholipid/fatty acid membrane encapsulating a pH clock reaction. We demonstrated that a simple pH change inside the vesicles lumen, coupled with an osmotic stress, is enough to induce shape transformations of the vesicles eventually leading to the fission of a mother GUV into two daughter GUVs [30,31]. Interestingly, our operative conditions are compatible with the slightly acidic pH suggested for the early Archean age oceans [57].

In this paper we presented some experiments that showed how the mechanical properties of the membrane are another ingredient necessary to understand the physical basis of protocells division. In fact, by varying the ratio between phospholipids and fatty acids, we could control both the ratio of the membrane’s bending moduli and the number of mobile molecules in the inner leaflet of the bilayer (i.e., the preferred area difference). We thus demonstrated that, for a mixed POPC/HOA vesicle, an effective budded shape could be attained only in a narrow range of the membrane composition, where the elasticity and the preferred area difference of the bilayer assume optimal values. According to experiments and simulations the budding region is comprised in the interval 0.8<α<1.2, which corresponds to 2<K<3 and $1.7<\Delta a_{0}^{\max}<1.9$.

## Supplementary Materials

The following are available online at https://www.mdpi.com/article/10.3390/life11070634/s1, Figure S1: (a) Frequency of divisions as a function of the sum [POPC] + [HOA] at a fixed a = [HOA]/[POPC] = 1; (b) Percentage of POPC in the final vesicles vs initial concentration of POPC in mineral oil during preparation. Data were extrapolated from HPLC analysis (chromatogram in the inset); error bars represent the standard deviation of three replicates, Table S1: Initial concen-trations and parameters used for the kinetic simulations, Table S2: Kinetic constants used in the model, Table S3: Initial conditions and parameters used for the case [POPC]: [HOA] = 2.6 mM:2.4.

## References

[1] Ludlow R.F., Otto S. (2008). Systems Chemistry. Chem. Soc. Rev..

[2] Ruiz-Mirazo K., Briones C., de la Escosura A. (2014). Prebiotic Systems Chemistry: New Perspectives for the Origins of Life. Chem. Rev..

[3] Ashkenasy G., Hermans T.M., Otto S., Taylor A.F. (2017). Systems Chemistry. Chem. Soc. Rev..

[4] Stano P., Rampioni G., D’Angelo F., Altamura E., Mavelli F., Marangoni R., Rossi F., Damiano L. (2018). Current Directions in Synthetic Cell Research. Advances in Bionanomaterials.

[5] Meng F., Zhong Z., Feijen J. (2009). Stimuli-Responsive Polymersomes for Programmed Drug Delivery. Biomacromolecules.

[6] Giuseppone N. (2012). Toward Self-Constructing Materials: A Systems Chemistry Approach. Acc. Chem. Res..

[7] Lopez A., Fiore M. (2019). Investigating Prebiotic Protocells for a Comprehensive Understanding of the Origins of Life: A Prebiotic Systems Chemistry Perspective. Life.

[8] Blain J.C., Szostak J.W. (2014). Progress Toward Synthetic Cells. Annu. Rev. Biochem..

[9] Buddingh’ B.C., van Hest J.C.M. (2017). Artificial Cells: Synthetic Compartments with Life-like Functionality and Adaptivity. Acc. Chem. Res..

[10] Rampioni G., Damiano L., Messina M., D’Angelo F., Leoni L., Stano P. (2013). Chemical Communication between Synthetic and Natural Cells: A Possible Experimental Design. Electron. Proc. Theor. Comput. Sci..

[11] Tomasi R., Noel J.M., Zenati A., Ristori S., Rossi F., Cabuil V., Kanoufi F., Abou-Hassan A. (2014). Chemical Communication between Liposomes Encapsulating a Chemical Oscillatory Reaction. Chem. Sci..

[12] Niederholtmeyer H., Chaggan C., Devaraj N.K. (2018). Communication and Quorum Sensing in Non-Living Mimics of Eukaryotic Cells. Nat. Commun..

[13] Aufinger L., Simmel F.C. (2019). Establishing Communication Between Artificial Cells. Chem. Eur. J..

[14] Budroni M.A., Torbensen K., Ristori S., Abou-Hassan A., Rossi F. (2020). Membrane Structure Drives Synchronization Patterns in Arrays of Diffusively Coupled Self-Oscillating Droplets. J. Phys. Chem. Lett..

[15] Altamura E., Milano F., Tangorra R.R., Trotta M., Omar O.H., Stano P., Mavelli F. (2017). Highly Oriented Photosynthetic Reaction Centers Generate a Proton Gradient in Synthetic Protocells. Proc. Natl. Acad. Sci. USA.

[16] Altamura E., Albanese P., Marotta R., Milano F., Fiore M., Trotta M., Stano P., Mavelli F. (2021). Chromatophores Efficiently Promote Light-Driven ATP Synthesis and DNA Transcription inside Hybrid Multicompartment Artificial Cells. Proc. Natl. Acad. Sci. USA.

[17] de Souza T.P., Steiniger F., Stano P., Fahr A., Luisi P.L. (2011). Spontaneous Crowding of Ribosomes and Proteins inside Vesicles: A Possible Mechanism for the Origin of Cell Metabolism. ChemBioChem.

[18] van Roekel H.W.H., Rosier B.J.H.M., Meijer L.H.H., Hilbers P.A.J., Markvoort A.J., Huck W.T.S., de Greef T.F.A. (2015). Programmable Chemical Reaction Networks: Emulating Regulatory Functions in Living Cells Using a Bottom-up Approach. Chem. Soc. Rev..

[19] Zhu T.F., Szostak J.W. (2009). Coupled growth and division of model protocell membranes. J. Am. Chem. Soc..

[20] Peterlin P., Arrigler V., Kogej K., Svetina S., Walde P. (2009). Growth and shape transformations of giant phospholipid vesicles upon interaction with an aqueous oleic acid suspension. Chem. Phys. Lipids.

[21] Kurihara K., Tamura M., Shohda K.I., Toyota T., Suzuki K., Sugawara T. (2011). Self-reproduction of supramolecular giant vesicles combined with the amplification of encapsulated DNA. Nat. Chem..

[22] Sakuma Y., Imai M. (2015). From Vesicles to Protocells: The Roles of Amphiphilic Molecules. Life.

[23] Jimbo T., Sakuma Y., Urakami N., Ziherl P., Imai M. (2016). Role of Inverse-Cone-Shape Lipids in Temperature-Controlled Self-Reproduction of Binary Vesicles. Biophys. J..

[24] Dervaux J., Noireaux V., Libchaber A.J. (2017). Growth and instability of a phospholipid vesicle in a bath of fatty acids. Eur. Phys. J. Plus.

[25] Litschel T., Ramm B., Maas R., Heymann M., Schwille P. (2018). Beating Vesicles: Encapsulated Protein Oscillations Cause Dynamic Membrane Deformations. Angew. Chem. Int. Ed..

[26] Kurisu M., Aoki H., Jimbo T., Sakuma Y., Imai M., Serrano-Luginbühl S., Walde P. (2019). Reproduction of vesicles coupled with a vesicle surface-confined enzymatic polymerisation. Commun. Chem..

[27] Li Y., ten Wolde P.R. (2019). Shape Transformations of Vesicles Induced by Swim Pressure. Phys. Rev. Lett..

[28] Vutukuri H.R., Hoore M., Abaurrea-Velasco C., van Buren L., Dutto A., Auth T., Fedosov D.A., Gompper G., Vermant J. (2020). Active particles induce large shape deformations in giant lipid vesicles. Nature.

[29] Dreher Y., Jahnke K., Bobkova E., Spatz J.P., Göpfrich K. (2021). Division and Regrowth of Phase-Separated Giant Unilamellar Vesicles. Angew. Chem. Int. Ed..

[30] Miele Y., Medveczky Z., Hollo G., Tegze B., Derenyi I., Horvolgyi Z., Altamura E., Lagzi I., Rossi F. (2020). Self-Division of Giant Vesicles Driven by an Internal Enzymatic Reaction. Chem. Sci..

[31] Holló G., Miele Y., Rossi F., Lagzi I. (2021). Shape Changes and Budding of Giant Vesicles Induced by an Internal Chemical Trigger: An Interplay between Osmosis and pH Change. Phys. Chem. Chem. Phys..

[32] Hu G., Pojman J.A., Scott S.K., Wrobel M.M., Taylor A.F. (2010). Base-Catalyzed Feedback in the Urea-Urease Reaction. J. Phys. Chem. B.

[33] Miele Y., Bánsági T., Taylor A., Stano P., Rossi F., Rossi F., Mavelli F., Stano P., Caivano D. (2016). Engineering Enzyme-Driven Dynamic Behaviour in Lipid Vesicles. Advances in Artificial Life, Evolutionary Computation and Systems Chemistry.

[34] Miele Y., Bánsági T., Taylor A., Rossi F., Piotto S., Rossi F., Concilio S., Reverchon E., Cattaneo G. (2018). Modelling Approach to Enzymatic pH Oscillators in Giant Lipid Vesicles. Advances in Bionanomaterials I.

[35] Svetina S., Žekš B. (1989). Membrane bending energy and shape determination of phospholipid vesicles and red blood cells. Eur. Biophys. J..

[36] Käs J., Sackmann E. (1991). Shape transitions and shape stability of giant phospholipid vesicles in pure water induced by area-to-volume changes. Biophys. J..

[37] Wiese W., Harbich W., Helfrich W. (1992). Budding of lipid bilayer vesicles and flat membranes. J. Phys. Condens. Matter.

[38] Miao L., Seifert U., Wortis M., Döbereiner H.G. (1994). Budding transitions of fluid-bilayer vesicles: The effect of area-difference elasticity. Phys. Rev. E.

[39] Seifert U. (1997). Configurations of fluid membranes and vesicles. Adv. Phys..

[40] Svetina S., Žekš B. (2002). Shape behavior of lipid vesicles as the basis of some cellular processes. Anat. Rec. Off. Publ. Am. Assoc. Anat..

[41] Heinrich V., Svetina S., Žekš B. (1993). Nonaxisymmetric vesicle shapes in a generalized bilayer-couple model and the transition between oblate and prolate axisymmetric shapes. Phys. Rev. E.

[42] Ikari K., Sakuma Y., Jimbo T., Kodama A., Imai M., Monnard P.A., Rasmussen S. (2015). Dynamics of fatty acid vesicles in response to pH stimuli. Soft Matter.

[43] Bian X., Litvinov S., Koumoutsakos P. (2020). Bending Models of Lipid Bilayer Membranes: Spontaneous Curvature and Area-Difference Elasticity. Comput. Methods Appl. Mech. Eng..

[44] Pautot S., Frisken B.J., Weitz D.A. (2003). Production of Unilamellar Vesicles Using an Inverted Emulsion. Langmuir.

[45] Carrara P., Stano P., Luisi P.L. (2012). Giant Vesicles Colonies: A Model for Primitive Cell Communities. ChemBioChem.

[46] Stano P., Wodlei F., Carrara P., Ristori S., Marchettini N., Rossi F., Pizzuti C., Spezzano G. (2014). Approaches to Molecular Communication Between Synthetic Compartments Based on Encapsulated Chemical Oscillators. Advances in Artificial Life and Evolutionary Computation.

[47] Fiore M., Maniti O., Girard-Egrot A., Monnard P.A., Strazewski P. (2018). Glass Microsphere-Supported Giant Vesicles for the Observation of Self-Reproduction of Lipid Boundaries. Angew. Chem. Int. Ed..

[48] Sakashita A., Urakami N., Ziherl P., Imai M. (2012). Three-dimensional analysis of lipid vesicle transformations. Soft Matter.

[49] Helfrich W. (1974). The size of bilayer vesicles generated by sonication. Phys. Lett. A.

[50] Brakke K.A. (1992). The Surface Evolver. Exp. Math..

[51] Carrara P. (2011). Constructing a Minimal Cell. Ph.D. Thesis.

[52] Tyler A.I.I., Greenfield J.L., Seddon J.M., Brooks N.J., Purushothaman S. (2019). Coupling Phase Behavior of Fatty Acid Containing Membranes to Membrane Bio-Mechanics. Front. Cell Dev. Biol..

[53] Kurniawan J., Suga K., Kuhl T.L. (2017). Interaction Forces and Membrane Charge Tunability: Oleic Acid Containing Membranes in Different pH Conditions. Biochim. Biophys. Acta (BBA) Biomembr..

[54] Majhenc J., Božič B., Svetina S., Žekš B. (2004). Phospholipid Membrane Bending as Assessed by the Shape Sequence of Giant Oblate Phospholipid Vesicles. Biochim. Biophys. Acta (BBA) Biomembr..

[55] Novák B., Tyson J.J. (2008). Design principles of biochemical oscillators. Nat. Rev. Mol. Cell Biol..

[56] Murtas G. (2013). Early self-reproduction, the emergence of division mechanisms in protocells. Mol. Biosyst..

[57] Halevy I., Bachan A. (2017). The Geologic History of Seawater pH. Science.

