# Supplementary material for: Effect of the Membrane Composition of Giant Unilamellar Vesicles on Their Budding Probability: A Trade-Off between Elasticity and Preferred Area Difference
Source: Life (Basel). 2021 Jun 29;11(7):634. doi: 10.3390/life11070634 (PMC8306738; doi:10.3390/life11070634)
Supplement: Supplementary file 1 [file life-11-00634-s001.zip › life-1276123-supplementary.pdf]

life

MDPI

Article

Supporting Information for: Effect of the Membrane Composition of Giant Unilamellar Vesicles on Their Budding Probability: a Trade-Off Between Elasticity and Preferred Area Difference

Ylenia Miele [1,‡], Gábor Holló [2,‡], István Lagzi [2,3,*] and Federico Rossi [4,*]

[1] Department of Chemistry and Biology "A. Zambelli", University of Salerno, Via Giovanni Paolo II 132, 84084 - Fisciano (SA), Italy; ymiele@unisa.it
[2] MTA-BME Condensed Matter Physics Research Group, Budapest University of Technology and Economics, Budafoki ut 8, Budapest - Hungary; hollo88@gmail.com, istvanlagzi@gmail.com
[3] Department of Physics, Budapest University of Technology and Economics 1111, Budafoki ut 8 - Budapest, Hungary;
[4] Department of Earth, Environmental and Physical Sciences - DEEP Sciences, University of Siena, Pian dei Mantellini 44, 53100 - Siena, Italy; federico.rossi2@unisi.it
* Correspondence: istvanlagzi@gmail.com; Tel.: +3614631341 (I.L.); federico.rossi2@unisi.it; Tel.: +390577232215 (F.R.)
‡ These authors contributed equally to this work.

check for updates

Citation: Miele, Y.; Hollo, G.; Lagzi, I.; Rossi, F. *Supporting Information for:* Effect of the Membrane Composition of Giant Unilamellar Vesicles on Their Budding Probability: a Compromise Between Elasticity and Preferred Area Difference. *Life* **2021**, *11*, 634. https://dx.doi.org/10.3390/life11070634

Academic Editors: Michele Fiore and Emiliano Altamura

Received: 10 June 2021
Accepted: 25 June 2021
Published: 29 June 2021

Publisher's Note: MDPI stays neutral with regard to jurisdictional claims in published maps and institutional affiliations.

Copyright: © 2021 by the authors. Licensee MDPI, Basel, Switzerland. This article is an open access article distributed under the terms and conditions of the Creative Commons Attribution (CC BY) license (https://creativecommons.org/licenses/by/4.0/).

1. Experimental: *Phase Transfer Method*

Hybrid giant vesicles were prepared using the droplet transfer technique with experimental conditions previously optimised [1,2]. Firstly, in an Eppendorf tube of 1.5 mL the outer solution was prepared: 300 μL of a solution of POPC and HOA dissolved in different proportions in mineral oil were poured over 500 μL of a water solution with glucose 0.2 mM. The entire set up was allowed to incubate for 10 minutes to form an hybrid monolayer. In a second Eppendorf tube, a water in oil microemulsion was prepared by hand pipetting a water phase with an apolar phase. The aqueous phase (20 μL) consisted of pyranine 50 μM, urease 0.5 U/mL, acetic acid 1 $\times 10^{-3}$mM and sucrose 200 mM. The apolar phase (600 μL) was made of HOA and POPC at different proportions in mineral oil. The microemulsion was poured over the first Eppendorf tube. The formation of vesicles was facilitated by centrifuging the tube at 6000 rpm for 10 minutes at room temperature. After the centrifugation step, a white pellet was visible at the bottom of the Eppendorf tube. The oil phase and the aqueous phase were carefully removed with a micropipette. The pellet was gently washed with 200 μL of O-solution to remove free solutes. 30 μL of pellet were diluted to 90 μL by adding O-solution.

2. ODE model

The reactions that take place in the hybrid POPC/HOA vesicles involve mainly 8 chemical species, namely urea (S, substrate), urease (E, enzyme), pyranine (fluorescent dye, pyrOH), oleic acid (HOA), acetic acid (HA), ammonia. The main equilibria are

$$CO(NH_2)_2 + H_2O \xrightarrow{urease} 2NH_3 + CO_2 \tag{1}$$

$$NH_4^+ \underset{}{\overset{k_2}{\rightleftarrows}} NH_3 + H^+ \tag{2}$$

$$CO_2 + H_2O \overset{k_3}{\rightleftarrows} H^+ + HCO_3^- \tag{3}$$

$$HCO_3^- \overset{k_4}{\rightleftarrows} H^+ + CO_3^{2-} \tag{4}$$

$$H_2O \overset{k_5}{\rightleftarrows} H^+ + OH^- \tag{5}$$

$$HA \overset{k_6}{\rightleftarrows} A^- + H^+ \tag{6}$$

$$HOA \overset{k_7}{\rightleftarrows} OA^- + H^+ \tag{7}$$

$$pyrOH \overset{k_8}{\rightleftarrows} pyrO^- + H^+ \tag{8}$$

The permeation of the neutral species (urea, ammonia, carbon dioxide and acetic acid) can be considered as a first order process described by the following equilibria

$$S \overset{k_S}{\rightleftharpoons} S_{out} \tag{9}$$

$$NH_3 \overset{k_N}{\rightleftharpoons} NH_{3out} \tag{10}$$

$$CO_2 \overset{k_C}{\rightleftharpoons} CO_{2out} \tag{11}$$

$$HA \overset{k_{HA}}{\rightleftharpoons} HA_{out} \tag{12}$$

where $[S]_{out}$, $[CO]_{2out}$, $[HA]_{out}$ and $[NH]_{3out}$ are respectively the concentration of urea, carbon dioxide, acetic acid and ammonia outside the vesicles in the water phase.
The pH change of the reaction affects the number of pH-sensitive amphiphilic molecules present in the bilayer (HOA and OA^-), while the number of POPC molecules is considered constant. The number of molecules in the outer leaflet is indicated as N_{outer}, the number of molecules in the inner leaflet is N_{inner}. The molecules present in the bilayer are POPC, HOA and OA^-. Thus the total number of molecules in the outer leaflet is $N_{outer} = N_{POPC\,outer} + N_{HOA\,outer} + N_{OA^-\,outer}$; the total number of molecules in the inner leaflet is $N_{inner} = N_{POPC\,inner} + N_{HOA\,inner} + N_{OA^-\,inner}$. Some oleic acid molecules in the inner leaflet can be deprotonated and solubilised as monomers or larger aggregates into the vesicle lumen ($N_{OA^-\,free\,inner}$ and $N_{HOA\,free\,inner}$); the loss of oleate is compensated by oleic acid molecules diffusing from the outer leaflet.

$$N_{HOA\,inner} \overset{k_7}{\rightleftharpoons} N_{OA^-\,inner} + H^+ \tag{13}$$

$$N_{OA^-\,inner} \overset{k_{off}}{\rightleftharpoons} N_{OA^-_{free\,inner}} \tag{14}$$

$$N_{HOA\,outer} \overset{k_f}{\rightleftharpoons} N_{HOA\,inner} \tag{15}$$

$$N_{HOA\,free\,inner} \overset{k_7}{\rightleftharpoons} N_{OA^-\,free\,inner} + H^+ \tag{16}$$

The set of ordinary differential equations (ODEs) derived from the equilibria 1-8, 9-12 and 13-16 is

$$\frac{d[S]}{dt} = -R + k_S([S]_{out} - [S]) \tag{17}$$

$$\frac{d[NH_3]}{dt} = 2R + k_2\left[NH_4^+\right] - k_{2r}[NH_3]\left[H^+\right] + k_N([NH_3]_{out} - [NH_3]) \tag{18}$$

$$\frac{d\left[NH_4^+\right]}{dt} = -k_2\left[NH_4^+\right] + k_{2r}[NH_3]\left[H^+\right] \tag{19}$$

$$\frac{d[CO_2]}{dt} = R - k_3[CO_2] + k_{3r}\left[H^+\right]\left[HCO_3^-\right] + k_C([CO_2]_{out} - [CO_2]) \tag{20}$$

$$\frac{d\left[HCO_3^-\right]}{dt} = k_3[CO_2] - k_{3r}\left[H^+\right]\left[HCO_3^-\right] - k_4\left[HCO_3^-\right] + k_{4r}\left[CO_3^{2-}\right]\left[H^+\right] \tag{21}$$

$$\frac{d\left[CO_3^{2-}\right]}{dt} = k_4\left[HCO_3^-\right] - k_{4r}\left[HCO_3^-\right]\left[H^+\right] \tag{22}$$

$$\begin{aligned}\frac{d[H^+]}{dt} = {} & k_2\left[NH_4^+\right] - k_{2r}[NH_3]\left[H^+\right] + k_3[CO_2] - k_{3r}\left[H^+\right]\left[HCO_3^-\right] + k_4\left[HCO_3^-\right] \\ & - k_{4r}\left[CO_3^{2-}\right]\left[H^+\right] + k_5 - k_{5r}\left[H^+\right]\left[OH^-\right] + k_6[HA] - k_{6r}\left[A^-\right]\left[H^+\right] + \\ & k_8[pyrOH] - k_{8r}\left[pyrO^-\right]\left[H^+\right] + k_7\frac{N_{innerHOA}}{N_{Av}V_p} - k_{7r}\frac{N_{innerOA^-}}{N_{Av}V_p}\left[H^+\right] \\ & + k_7\frac{N_{HOA\,free\,inner}}{N_{Av}V_p} - k_{7r}\frac{N_{OA^-\,free\,inner}}{N_{Av}V_p}\left[H^+\right]\end{aligned} \tag{23}$$

$$\frac{d[OH^-]}{dt} = k_5 - k_{5r}\left[H^+\right]\left[OH^-\right] \tag{24}$$

$$\frac{d[HA]}{dt} = -k_6[HA] + k_{6r}\left[A^-\right]\left[H^+\right] + k_{HA}([HA]_{out} - [HA]) \tag{25}$$

$$\frac{d[A^-]}{dt} = k_6[HA] - k_{6r}\left[A^-\right]\left[H^+\right] \tag{26}$$

$$\frac{d[pyrOH]}{dt} = -k_8[pyrOH] + k_{8r}\left[pyrO^-\right]\left[H^+\right] \tag{27}$$

$$\frac{d[pyrO^-]}{dt} = k_8[pyrOH] - k_{8r}\left[pyrO^-\right]\left[H^+\right] \tag{28}$$

$$\frac{dN_{outer}}{dt} = -k_f\left(N_{outer} - N_{inner}\frac{R_s^2}{(R_s-h)^2}\right) \tag{29}$$

$$\frac{dN_{inner}}{dt} = +k_f\left(N_{outer} - N_{inner}\frac{R_s^2}{(R_s-h)^2}\right) - N_{innerOA^-}\frac{k_{off}}{1+e^{(-k_t\,(pH-pH_{thres}))}} \tag{30}$$

R is a modified Michaelis-Menten rate law, which accounts for the pH dependence, the substrate and the product inhibition of the enzyme

$$R = \frac{v_{max}\,[S]}{\left(K_M + [S]\left(1+\frac{[S]}{K_S}\right)\left(1+\frac{[P]}{K_P}\right)\left(1+\frac{K_{es2}}{[H^+]}+\frac{[H^+]}{K_{es1}}\right)\right)} \tag{31}$$

N_{Av} is the Avogadro's number and V_p is an average volume calculated from the prolate and the pear shapes expressed in dm^3 (for simplicity, the volume is kept constant during the transformation from the prolate to the budded limiting shape). The equations contain concentrations (indicated in square brackets) and numbers of molecules (N_{outer}, N_{outer}, N_{inner}, $N_{innerOA^-}$, $N_{OA^-\,free\,inner}$ and $N_{HOA\,free\,inner}$). The transport of oleate molecules from the outer leaflet to the inner leaflet occurs with a reaction rate that takes into account the geometry of the vesicle; the dissolution of oleate from the inner leaflet to the aqueous

pool occurs with a rate that depends on the pH of the solution. The logistic function was implemented in the model described in ref. [2]. Concentrations and numbers of molecules are correlated through the Avogadro's number and the volume of the vesicles.
The initial total number of molecules for the outer (N_{outer}) and the inner leaflet (N_{inner}) was calculated from the spherical vesicle:

$$N_{\text{outer}} = \frac{4\pi R_s^2}{< \tilde{a} >} \tag{32}$$

$$N_{\text{inner}} = \frac{4\pi (R_s - h)^2}{< \tilde{a} >} \tag{33}$$

where $< \tilde{a} >$ is the mean cross-sectional area expressed in nm^2 and calculated as reported in ref. [2]. The number of molecules of POPC and Oleic acid present in membrane was derived from the initial composition.

$$N_{\text{outer}} = N_{\text{outerPOPC}} + N_{\text{outerOleic}} \tag{34}$$

$$N_{\text{outerPOPC}} = \frac{N_{\text{outer}}}{1 + \frac{[\text{Oleic}]_0}{[\text{POPC}]_0}} \tag{35}$$

$$N_{\text{outerOleic}} = N_{\text{outer}} - N_{\text{outerPOPC}} \tag{36}$$

The initial concentrations and parameters used for the simulations are reported in Table 1. The kinetic constants related to the equilibria 1-8 and 9-12 are listed in Table 2. The kinetic constants for the equilibria of HOA and OA^-(eq. 13-16) are listed in Table 3.

Table 1: Initial concentrations and parameters used for the kinetic simulations. $[CO_2]$ and $[CO_2]_{out}$ are calculated by considering the solution at the equilibrium with the atmosphere at 25 °C. The transfer rates k_X (s^{-1}) were calculated from the permeabilities P_X as $k_X = 3P_X/R$ where R (dm) is the vesicle radius.

[X] (M)		Parameters	
[S]	0	[E] (U/mL)	1.10
$[NH_3]$	0	$[S]_{out}$ (M)	6.00×10^{-2}
$[NH_4^+]$	0	$[NH_3]_{out}$ (M)	0
$[CO_2]$	1.20×10^{-5}	$[H^+]_{out}$ (M)	1.00×10^{-6}
$[HCO_3^-]$	5.62×10^{-6}	$[OH^-]_{out}$ (M)	1.00×10^{-8}
$[CO_3^{2-}]$	3.15×10^{-10}	$[HA]_{out}$ (M)	5.45×10^{-8}
$[H^+]$	1.00×10^{-6}	$[CO_2]_{out}$ (M)	1.20×10^{-5}
$[OH^-]$	1.00×10^{-8}	P_S (dm/s) [3]	4.00×10^{-7}
[HA]	5.45×10^{-8}	P_N (dm/s) [4]	1.00×10^{-3}
$[A^-]$	9.45×10^{-7}	P_{HA} (dm/s) [5]	6.50×10^{-4}
[pyrOH]	4.81×10^{-5}	P_C (dm/s) [6]	1.20
$[pyrO^-]$	1.92×10^{-6}	V_P (dm^3)	6.82×10^{-13}

Table 2: Kinetic constants used in the model. Enzymatic constants were taken from refs. [7–9]. Equilibrium rate constants were derived from the pK_a according to refs. [9–11].

Enzymatic			pH equilibria	
k_1 (U^{-1} mL M s^{-1})	3.7 $\times 10^{-6}$		forward (s^{-1})	reverse (M^{-1} s^{-1})
K_m (M)	3.0 $\times 10^{-3}$	k_2	24	4.3 $\times 10^{10}$
K_{es1} (M)	5.0 $\times 10^{-6}$	k_3	3.7 $\times 10^{-2}$	7.9 $\times 10^{4}$
K_{es2} (M)	2.0 $\times 10^{-9}$	k_4	2.8	5 $\times 10^{10}$
K_S (M)	3.0	k_5	1$\times 10^{-3}$ (M^{-1} s^{-1})	1 $\times 10^{11}$
K_P (M)	2.0 $\times 10^{-3}$	k_6	7.8 $\times 10^{5}$	4.5 $\times 10^{10}$
		k_7	3.2 $\times$ 10^2	1 $\times 10^{10}$
		k_8	1	2.5 $\times 10^{7}$

Table 3: Initial conditions and parameters used for the case [POPC]: [HOA]=2.6 mM:2.4 mM.

Initial conditions		Parameters	
$N_{outerHOA}$	5.795 $\times$ 10^8	$N_{outerPOPC}$	6.476 $\times$ 10^8
$N_{outerOA^-}$	1.832 $\times$ 10^7	$N_{innerPOPC}$	6.473 $\times$ 10^8
$N_{innerHOA}$	5.792 $\times$ 10^8	R_S (nm)	7.12 $\times$ 10^3
$N_{innerOA^-}$	1.831 $\times$ 10^7	h (nm)	2
$N_{HOA\ free\ inner}$	0	k_f (s^{-1})	0.4
$N_{OA^-\ free\ inner}$	0	k_{off} (s^{-1})	0.008
		k_t	100
		pH_{thres}	6.3
		$< \tilde{a}(nm^2) >$	0.512

3. Dependence of division on *s* and HPLC Analysis

(*i*) Figure 1 reports the dependence of the division probability with respect to the total amount of amphiphiles in the initial solutions used for the vesicles generation. *s* was varied in the interval 0.3 mM – 10 mM with a fixed ratio [HOA]/[POPC] = 1 and the division frequency was found substantially independent from the total initial concentration of the amphiphilic molecules.

(*ii*) To quantify the average amphiphilic mass in a pellet, GUVs with different initial amphiphilic concentration were analysed by HPLC and a typical chromatogram is shown in the inset of Figure 1b. Figure 1b shows that the droplet transfer method yield do not change for the interval 0.3 < [POPC] < 1.0 mM (the percentage of POPC in the final vesicles is 2–4%), therefore the increase of the initial total concentration could have no effects on the membrane composition.

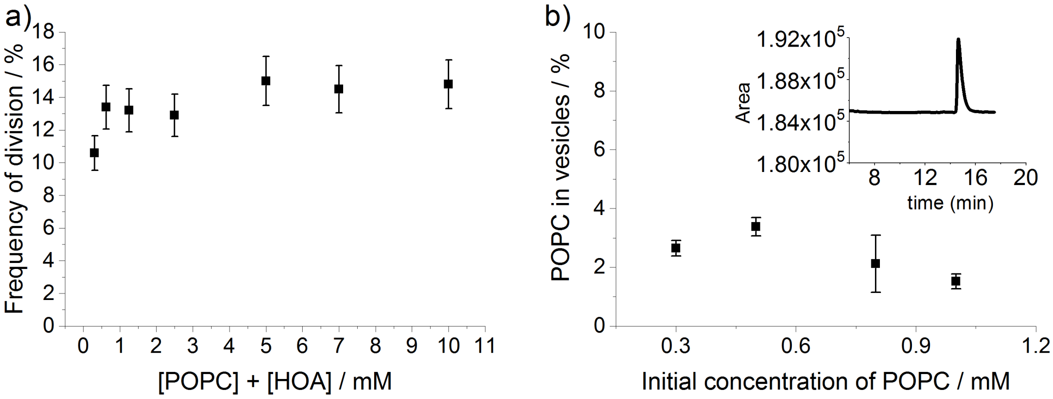

Figure 1. a) Frequency of divisions as a function of the sum [POPC] + [HOA] at a fixed α = [HOA]/[POPC] = 1; b) Percentage of POPC in the final vesicles vs initial concentration of POPC in mineral oil during preparation. Data were extrapolated from HPLC analysis (chromatogram in the inset); error bars represent the standard deviation of three replicates.

References

1. Miele, Y.; Medveczky, Z.; Hollo, G.; Tegze, B.; Derenyi, I.; Horvolgyi, Z.; Altamura, E.; Lagzi, I.; Rossi, F. Self-Division of Giant Vesicles Driven by an Internal Enzymatic Reaction. *Chemical Science* **2020**, *11*, 3228–3235. doi:10.1039/C9SC05195C.
2. Holló, G.; Miele, Y.; Rossi, F.; Lagzi, I. Shape Changes and Budding of Giant Vesicles Induced by an Internal Chemical Trigger: An Interplay between Osmosis and pH Change. *Physical Chemistry Chemical Physics* **2021**, *23*, 4262–4270. doi:10.1039/D0CP05952H.
3. Walter, A.; Gutknecht, J. Permeability of small nonelectrolytes through lipid bilayer membranes. *The Journal of membrane biology* **1986**, *90*, 207–217.
4. Lande, M.B.; Donovan, J.M.; Zeidel, M.L. The relationship between membrane fluidity and permeabilities to water, solutes, ammonia, and protons. *The Journal of general physiology* **1995**, *106*, 67–84.
5. Xiang, T.X.; Anderson, B.D. Permeability of acetic acid across gel and liquid-crystalline lipid bilayers conforms to free-surface-area theory. *Biophysical journal* **1997**, *72*, 223–237.
6. Missner, A.; Kügler, P.; Saparov, S.M.; Sommer, K.; Mathai, J.C.; Zeidel, M.L.; Pohl, P. Carbon dioxide transport through membranes. *Journal of Biological Chemistry* **2008**, *283*, 25340–25347.
7. Fidaleo, M.; Lavecchia, R. Kinetic study of enzymatic urea hydrolysis in the pH range 4-9. *Chemical and biochemical engineering quarterly* **2003**, *17*, 311–318.
8. Krajewska, B.; Ciurli, S. Jack bean (Canavalia ensiformis) urease. Probing acid–base groups of the active site by pH variation. *Plant Physiology and Biochemistry* **2005**, *43*, 651–658.
9. Hu, G.; Pojman, J.A.; Scott, S.K.; Wrobel, M.M.; Taylor, A.F. Base-Catalyzed Feedback in the Urea-Urease Reaction. *The Journal of Physical Chemistry B* **2010**, *114*, 14059–14063.
10. Eigen, M. Proton transfer, acid-base catalysis, and enzymatic hydrolysis. Part I: elementary processes. *Angewandte Chemie International Edition in English* **1964**, *3*, 1–19.
11. Wang, X.; Conway, W.; Burns, R.; McCann, N.; Maeder, M. Comprehensive study of the hydration and dehydration reactions of carbon dioxide in aqueous solution. *The journal of physical chemistry A* **2009**, *114*, 1734–1740.
